# An in-depth exploration of the post-test informational needs of *BRCA1* and *BRCA2* pathogenic variant carriers in Asia

**DOI:** 10.1186/s13053-020-00154-x

**Published:** 2020-10-23

**Authors:** Jeanette Yuen, Si Ming Fung, Chin Leong Sia, Mallika Venkatramani, Tarryn Shaw, Eliza Courtney, Shao-Tzu Li, Jianbang Chiang, Veronique Kiak-Mien Tan, Benita Kiat-Tee Tan, Joanne Ngeow

**Affiliations:** 1 Cancer Genetics Service, Division of Medical Oncology, National Cancer Centre Singapore, Singapore, Singapore; 2 Lee Kong Chian School of Medicine, Nanyang Technological University, 11 Mandalay Drive, Singapore, Singapore; 3 Singhealth-Duke NUS Breast Centre, Singapore, Singapore; 4 Department of Breast Surgery, Singapore General Hospital, Singapore, Singapore; 5 Department of Breast Surgery, National Cancer Centre Singapore, Singapore, Singapore; 6 Department of General Surgery, Sengkang General Hospital, Singapore, Singapore; 7 Oncology Academic Clinical Program, Duke-NUS Medical School, Singapore, Singapore

**Keywords:** *BRCA1/2*, Informational needs, Interview, Online, Genetic awareness

## Abstract

**Introduction:**

Identification of one’s status as a *BRCA1/2* pathogenic variant carrier often marks the start of navigating challenging decisions related to cancer risk management and result disclosure. Carriers report unmet informational needs, but studies have yet to explore the specific aspects of and how best to fulfill these needs. This study aims to explore the informational needs of *BRCA1/2* pathogenic variant carriers in Asia to inform for the design of educational materials to support risk management decision-making.

**Methods:**

Semi-structured in-depth interviews were conducted with two male and 22 female English-speaking *BRCA1/2* pathogenic variant carriers, aged 29–66 years, identified through the Cancer Genetics Service at the National Cancer Centre Singapore. A grounded theory approach with thematic analysis was undertaken to extract dominant themes.

**Results:**

Four themes were identified: (i) proactive online information seeking behaviors (ii) personalized informational needs; (iii) challenges in sharing the results; and (iv) lack of genetic awareness.

**Discussion:**

Participants highlight challenges with sharing their result arising from significant post-result informational needs, which have manifested into proactive online information-seeking behaviors. They desire for an online source of information, where content is personalized, reliable and local. Participants foresee the potential of an online resource to raise genetic awareness. This suggests the use of a culturally tailored online-based genetics resource, to promote result disclosure, empower risk-management decisions and raise genetic literacy rates.

**Supplementary information:**

**Supplementary information** accompanies this paper at 10.1186/s13053-020-00154-x.

## Introduction

Identification of individuals who carry pathogenic/likely pathogenic variants (PV/LPV) in *BRCA1/2* genes has improved our ability to manage, support, and counsel individuals and their family members who are at an increased risk of cancer [1, 2]. Female carriers of a monoallelic *BRCA1/2* PV/LPV have a 40–66% lifetime risk of developing breast cancer and a 13–46% lifetime risk of ovarian cancer [3, 4]. Male monoallelic *BRCA1/2* PV/LPV carriers face a 9–15% lifetime risk of developing prostate cancer and up to 7% lifetime risk of male breast cancer [5]. The actionable advantages of knowing one’s genetic status, which can be used to guide treatment, early detection, and risk-reducing (RR) strategies, have prompted support for the universal *BRCA1/2* testing of all breast cancer patients [6, 7].

Understanding one’s *BRCA1/2* status often marks the start of navigating challenging decisions. Monoallelic PV/LPV carriers need to consider their options for screening, chemoprevention, and RR surgery [8, 9] proven to reduce cancer risk and overall cancer mortality [10, 11]. They are next faced with the task of communicating their genetic test (GT) result to at-risk relatives, with challenges that differ by age, gender, and life stage [12–14].

Existing research reports that the highest unmet need of *BRCA1/2* PV/LPV carriers is information [15–17], yet few studies have explored the specific aspects of and how best to fulfill these needs [15, 18]. First, Augestad et al. [15] observed that *BRCA1/2* carriers found it difficult to digest and retain the information provided verbally at their GT result appointment, suggesting that the provision of information following this should be optimized to promote result adaptation. Babb et al. [18] found almost half of the *BRCA1/2* PV/LPV carriers they interviewed had unmet informational needs regarding subsequent risk management decisions to undergo RR bilateral salpingo-oophorectomy (RRBSO). They also reported difficulties in locating information, which became a source of frustration and anxiety. Furthermore, the general lack of qualitative data highlights the need to identify informational gaps that *BRCA1/2* PV/LPV carriers may encounter in medical, financial, social, and legal aspects [19] that presently go unrecognized.

Breast cancer continues to be one of the most common cancers that afflicts Asian women, where its incidence in Asia has increasingly surpassed the historically high rates in the United States [20]. Studies have also observed a greater proportion of young-onset (under age 40) breast cancer in Asia as compared to Western and European populations [21, 22], Therefore, as evidence for gene-directed treatments and risk management increases, it has resulted in an exponential growth for the demand for cancer genetic services in Asia [23]. Meeting the demand for genetic testing in Asia is not without its challenges, with cost barriers arising from the absence of adoption into national healthcare systems and poor genetic awareness of the public. Nonetheless, Asian populations are reporting higher detection rates of *BRCA1/2* PV/LPV carriers; as high as 25% in Asian females with breast cancer [23–25]. Locally, we have observed that adherence to breast cancer risk management of *BRCA1/2* PV/LPV carriers is much higher than ovarian cancer risk management [26] – warranting further investigation into the support and informational needs of carriers to make risk management decisions. The paucity of such information impedes the care of such at-risk individuals, as evidence-based improvements cannot be implemented to promote result adaption and empower risk management decision-making.

Therefore, an in-depth interview study was designed to explore the post-result informational needs of *BRCA1/2* male and female carriers in our Asian population, which has largely gone unstudied. This study aims primarily to identify specific informational needs of *BRCA1/2* PV/LPV carriers in Asia, and explore patient preferences to address these needs. Our findings would inform the delivery, design, and content of educational materials and informational support systems for *BRCA1/2* PV/LPV carriers to support risk management decision-making.

## Methods

### Participants

**Fig. 1:**
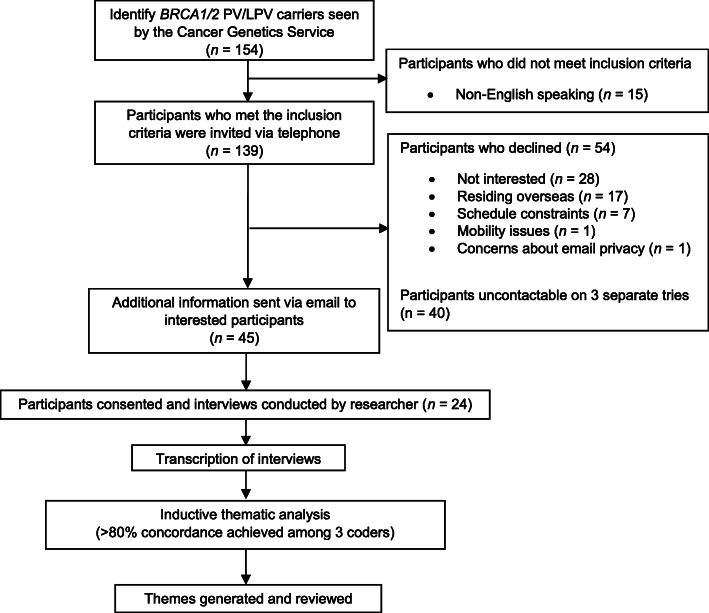
Flow chart of study design

Male and female *BRCA1/2* PV/LPV carriers who met the following criteria were included in the study [1]: English-speaking [2]; aged 25 and above; and [3] received genetic counselling through the Cancer Genetics Service (CGS) of the National Cancer Centre Singapore (NCCS). Purposive sampling was conducted, and participants were invited through a combination of telephone and email methods until data saturation was reached [27]. Arrangements were made to meet consented participants in-person to conduct the interview (Fig. 1). This qualitative study was approved by the Singapore Health Services Centralized Institutional Review Board (CIRB2018/3010). All participants provided written informed consent.

### Management of *BRCA1/2* PV/LPV carriers

All *BRCA1/2* PV/LPV carriers identified in this study were seen at the CGS for a pre-test genetic counselling session. This typically involved an in-person verbal discussion, with the use of visual aids, to provide an assessment of the a priori likelihood and information regarding the potential implications of identifying a *BRCA1/2* PV/LPV based on personal/family history. After consent to proceed with genetic testing was obtained, a second appointment was arranged for results disclosure, and to discuss the implications in greater detail (results appointment). Cancer risk management recommendations were discussed and necessary referrals to a breast surgeon and/or a gynecological oncologist were made. Appointments with these risk management specialists and annual follow-ups scheduled with the CGS are considered post-result consultations. Annual follow-ups with CGS were scheduled to monitor adherence to recommended risk-management and to encourage predictive testing of at-risk family members.

### Interviews

Interviews were semi-structured and included questions regarding interviewees’ opinions about the information received, informational gaps, and post-result challenges they faced. The interview guide (Supplementary Materials and Methods) was designed after a review of current literature by Masters-trained genetic counsellors with interview experience (JY; TS). Interviews were conducted by an experienced researcher (SMF) who had no previous interaction with the participants. The interviews were conducted in a private room, which lasted between 45 min to an hour.

### Data analysis

All interviews were audio-recorded. They were transcribed verbatim and coded using the NVivo Pro v.12 software. A grounded theory approach with inductive thematic analysis [28] was undertaken to facilitate the comprehensive exploration of the transcripts. All transcripts were coded by two independent coders (JY; SMF), any discordance was resolved by a third-party coder (CLS), ensuring > 80% coding concordance. Recurring codes were analyzed and reviewed with the research team iteratively to evaluate for theme generation and data saturation.

## Results

### Participants

One hundred and thirty-nine eligible *BRCA1/2* PV/LPV carriers were invited to participate in the interview. Data saturation was reached when 24 participants completed the interview (17% response rate) between August and September 2019 (Fig. 1). There were 11 (46%) *BRCA1* and 13 (54%) *BRCA2* monoallelic PV/LPV carriers. Most of the participants were female (91%), were married (54%), have children (62%), and were aged between 29 and 66 years (Table 1). Majority of the participants have a personal history of *BRCA1/2*-related cancer(s) (70%), a family history of cancer (91%) and had recently (< 1 year) learned their positive *BRCA1/2* PV/LPV carrier status (45%), with a median of 1.8 years since GT result disclosure.

**Table 1:** Participant characteristics

Characteristics		*N* = 24 (%)	Median
Age, years	25–30s	7 (29.2)	48.5
	40s	8 (33.3)	
	50s	6 (25.0)	
	60s	3 (12.5)	
Time since result disclosure, year	Less than 1 year	11 (45.8)	1.8
	1–2 years	2 (8.3)	
	2–3 years	4 (16.7)	
	3–4 years	6 (25.0)	
Sex	Female	22 (91.6)	–
	Male	2 (8.3)	
Race	Chinese	15 (62.5)	–
	Malay	3 (12.5)	
	Indian	2 (8.3)	
	Others	4 (16.7)	
Marital status	Married	13 (54.2)	–
	Single	8 (33.3)	
	Divorced	2 (8.3)	
	Widowed	1 (4.2)	
Children	Yes	15 (62.5)	–
	No	9 (37.5)	
Employment status	Full-time	13 (54.2)	–
	Part-time	6 (25.0)	
	Unemployed	5 (20.8)	
Education level	Primary	1 (4.2)	–
	Secondary	5 (20.8)	
	Polytechnic^*a*^	4 (16.7)	
	Graduate	9 (37.5)	
	Postgraduate	5 (20.8)	
Individual Monthly income (SGD)	< 2500	6 (25.0)	–
	2500–5000	5 (20.8)	
	5000–7500	3 (12.5)	
	> 7500	7 (29.2)	
	Missing/ NIL	3 (12.5)	
Received financial assistance for genetic testing	Yes	10 (41.7)	–
	No	14 (58.3)	
Personal history of breast, ovarian, or prostate cancer	Yes	17 (70.8)	–
	No	7 (29.2)	
Family history of cancer	Yes	22 (91.7)	–
	No	1 (4.2)	
	Unknown	1 (4.2)	

^*a*^Institution of higher education offering courses at degree level or below, especially in vocational subjects

Four major themes were identified relating to informational gaps and post-result challenges faced by participants. Firstly, participants highlight (i) *challenges in sharing their result* as an outcome of poor personal understanding and informational support. As a result of significant informational needs, carriers practice (ii) *proactive online information-seeking behaviors* which comes with its own set of challenges. Participants have vocalized the preference for (iii) *personalized information* to address information gaps. Lastly, carriers lament the (iv) *lack of genetic awareness* which results in poor societal, and therefore, familial understanding of the implications of their result and advocate for greater publicity of genetic concepts.

**Challenges in sharing** the **results*:*** *“I wouldn’t have the knowledge to really explain”.*

The majority of participants (21/24) reported challenges in sharing their GT result and its implications with at-risk relatives and/or friends as a result of not having sufficient information. Most of the participants (19/24) highlighted questions raised by their family which they were unable to answer. Of which, some (11/21) have dealt with negative reactions of shock, worry, and skepticism from their family members.

*"Because if they were to ask, “What is this BRCA1?” I wouldn’t have the knowledge to really explain to them. They wouldn’t really understand. The result also … if I were to show to my family members, they say, “Wah, so cheem (Singaporean slang for complex), what is this?” They won’t really understand. So maybe there’s a video or pamphlet that give more understanding in simpler words. Maybe they can grasp this’ –* 49-year-old female, *BRCA1* PV/LPV carrier, with breast cancer.

Participants aged 40 and below (4/7) also reported a set of challenges specific to communicating their GT result to their young children. A 34-year-old female (*BRCA2* PV/LPV carrier) with breast cancer explained that *“knowing how to explain in a kid-friendly way will be helpful”,* as she has a young daughter who finds it “*hard to grasp*, *so instead of understanding, sometimes the fear takes over”.* Other carriers also report challenges which include having to justify why they undergo early screening and/or RR surgery to their friends and family.

*“Some wouldn’t understand why I would do prophylactic surgery. I think that is the challenge. To some people the risk is not high, to some it’s very high, so I think it’s a very varied response, so not everybody will understand why we make such decisions. I did my oophorectomy and … there’re still people who’ll are like, ‘Oh, why on earth did you remove your ovaries, because your risk is not that high’” –* 45-year-old unaffected female, *BRCA2* PV/LPV carrier.

Participants (7/24) have suggested strategies that could facilitate the process of communicating their GT result with at-risk family members. Their ideas largely focused on the provision of accessible, visual content that is easily shared, obliterating the need for an in-person clinic consultation to understand why testing for them could be useful.

*“Maybe a video or something, that you know, easily accessible. Like my siblings, you know they one in denial, another one he say, “Ah, won’t get it” ... So maybe if there’s a video, then they can just, click to it, link to it, then maybe can see on their own time, maybe it’s easier for them. Because for them to come here, I don’t think so they would”* – 49-year-old female, *BRCA1* PV/LPV carrier, with breast cancer.

### Proactive online information seeking behaviors: *“people will Google for sure”*

As an outcome from their need for additional information to supplement to their understanding of what it means to be a *BRCA1/2* PV/LPV carrier, many participants (15/24) proactively sought online sources of information using Internet search engines.

Whilst online sources provided additional information, the same 15 participants reported challenges with online information, which included a lack of balanced information and local population data. A 45-year-old unaffected female (*BRCA2* PV/LPV carrier) found “*a lot of patient information groups, mainly US-based … I couldn’t find anything from Singapore per se” and* noted that because *“the US guidelines are a bit slanted towards surgery”,* this made it *“very hard to find a very balanced opinion”* on the advantages and disadvantages of undergoing RR mastectomy.

Furthermore, several of them (7/15 online information seekers) highlighted the dangers of reading unreliable and unregulated websites, which may provide conflicting and potentially inaccurate information. One-third of them (5/15 online information seekers) also noted that sharing of patients’ stories in this context would be helpful in the decision-making process for risk management.

*“You can say, you have this patient, and these are the decisions she made based on that, I mean I feel If you give an example of a patient who went through it and the decisions that patients made, why or whatever, you can relate more easily, rather than just a statistic*” – 46 year-old female, *BRCA1* PV/LPV carrier, with breast cancer.

Most of them (11/15 online information seekers) provided strategies on how to solve the challenges of online information, which included the need for clinicians and/or genetic services to provide sources of reliable online information, or the direct provision of a take-home *“resource pack”* outlining potential services to support recommended risk management.

*“Because people will Google for sure ... So, if NCC (National Cancer Centre) has a list of websites that they feel are very useful or more dependable, then that would be good to share with the patient” –* 45-year-old unaffected female, *BRCA2* PV/LPV carrier.

**Personalized informational needs: “***what it means for me personally”.*

Content-wise, many participants (17/24) expressed the desire for information to be personalized; relevant based different attributes like their personal and family history of cancer, age, and experience with surgery.

*“It will be helpful to know what it means for me personally, ‘cause it’s very easy to talk about this demographic but sometimes I don’t really fit there you know … So I think a more personalized, holistic approach for each person … that’s the challenge”* – 56 year-old female, *BRCA1* PV/LPV carrier, with ovarian cancer.

In our study cohort, most of the participants (17/24) were aged over 40 years old, while the remaining (7/24) were aged 40 and under. Participants advocated for the provision of information tailored to their age (11/17 over 40; 6/7 aged 40 and under; 17/24). Similarly, participants (4/7 unaffected carriers; 10/17 affected carriers; 14/24) preferred for information to be personalized based on their cancer status.

*“The difference between knowing as a cancer patient and knowing as an unaffected family member … that’s different also, you want to cater your information then, as it is quite different groups of people that you are looking at, ‘cause the implications of knowing are slightly larger if you are someone who has not been diagnosed with cancer before”* – 32-year-old female, *BRCA2* PV/LPV carrier, with breast cancer.

Specific to understanding the implications of being a *BRCA1/2* PV/LPV carrier, older participants expressed different forms of misunderstandings from younger counterparts. Of the 10 participants with misconceptions of their *BRCA1/2* result, nine of them were aged over 40 years. They misunderstood different implications of their GT result: it affects cancer relapse or metastasis; only affects females; it will inevitably be passed down to their children.

Of the 15 participants who needed more online information to support their understanding of their carrier status, 11 of them were aged over 40 years. However, distinct from their younger counterparts, they tended to request for more layman information specific to the content of their GT report and how accurate their results are.

*“The report that comes out is quite complex … but for the layperson to understand … there is a question … For me even the start would be: what is BRCA1, what is that gene? How do you detect it? Um, what is this variant?*” – 59-year-old unaffected male, *BRCA1* PV/LPV carrier.

Most of those over age 40 (11/17) had expressed their desire for clinicians to provide tools such as visual aids, or take-home information booklets to guide and facilitate supportive discussions to promote better comprehension during their GT result appointment and consultations with their risk management specialists (breast/gynecology).

*“I think the doctor actually [can] take effort, in explaining clearly … maybe some visual aids [can] help, because what stuck in my head was that graph thing... I think pictures or graphs help people to remember better” –* 43-year-old female, *BRCA1* PV/LPV carrier, with breast cancer.

All of the participants aged 40 and below (7/7) requested for more information specific to support their decision-making process regarding surveillance or RR surgery. They raised gaps in their understanding of real-life implications associated with RR mastectomies and/or RRBSO, such as its impact on sexual life and marriage, the logistics of surgery, the associated recovery time and its impact on their quality of life.

*"I think for a young woman, you ask them to chop off your breast, it’s not exactly an easy thing to do immediately. I think, implications on my sexual health as well, when I think about removing my ovaries in the future, for my husband’s sake, even though he will of course say that take care of yourself first, that might be physical but [it has] effects on other aspects as well. –* 32-year-old female, *BRCA2* PV/LPV carrier, without children, with breast cancer.

All the participants aged 40 and below (7/7) highlighted ways that they would like to be supported as well, which varied greatly from participants aged over 40. They raised the idea of formal information support systems by way of having a designated person to discuss any questions they may encounter along the way with or matching them with other *BRCA1/2* PV/LPV carriers who are age-compatible to be able to work through some of the issues they face.

*“Cause sometimes you might feel a bit stupid asking your doctor lifestyle questions I guess … so somebody that might be able to tell you a little bit about how it affects everything other than physically. And again, because there’s that element of … what I learn through Facebook forums and 9 out of 10 of them are having a very bad experience, so somebody that could reassure me that it [surgical menopause] doesn’t necessarily have to be awful”* – 38-year-old unaffected female, *BRCA2* PV/LPV carrier.

**Lack of genetic awareness:** *“more people should know”.*

The majority of participants (18/24) highlighted a lack of awareness regarding genetic testing, hereditary cancer and subsequent recommended cancer risk management options (i.e. early screening or RR surgery) not only in the general public but also among their healthcare providers.

A 49-year-old unaffected female (*BRCA2* PV/LPV carrier) with a strong family history of *BRCA1/2*-associated cancers lamented that it took almost nine years of *“actively searching”* before a doctor told her of the availability of genetic testing*.* On knowing her GT result, her original gynecologist *“seemed quite surprised that there actually is such a thing, and you can do genetic testing”.* This participant hopes to see *“one improvement to be made … that more doctors are familiar and then they can give advice”.*

Half of the participants (12/24) also raised several advantages with increased awareness and genetic literacy across the society, it would: increase the uptake of genetic testing among at-risk individuals; facilitate the communication of a positive GT result and its implications to at-risk relatives; reduce the stigma and discrimination associated with having a genetic predisposition to cancer and their decision to undergo RR surgeries.

*“More people should know, like we knew about this BRCA thing only because of Angelina Jolie’s whole episode but … people shouldn’t be scared of talking about it and people should even be told to please tell their family members about it, there shouldn’t be a stigma” –* 46-year-old female, *BRCA1* PV/LPV carrier, with breast cancer.

It was also highlighted that greater awareness of the availability of genetic testing and its clinical implications of a positive GT result could also improve the way insurance coverage is made available to consumers. All the unaffected carriers (7/24) expressed concern over possible discrimination of themselves and their family members by insurance companies and worry that previously purchased coverage would not cover RR surgeries. This is aligned with cost concerns that many participants (17/24) have raised, relating to the high cost of genetic testing and subsequent fees associated screening, surveillance and RR surgeries.

*“The genetic test is actually very expensive. So, actually, if you want to really help people with this, the genetic test needs to be cheaper, or subsidized, because right now it is not”* – 51-year-old female, *BRCA1* PV/LPV carrier, with breast cancer.

Majority of the participants (20/24) suggested strategies to raise genetic awareness, which largely included the sharing of local stories and the use of mass media (i.e. online videos, social media) to gain more public awareness.

*“If there’s an Angelina Jolie from Caldecott Hill (Singapore’s version of Hollywood). I think she did … more for raising awareness than anyone else could ever have done... But I think it’s helpful to have local stories and local examples because people still view it a bit differently as a Caucasian disease” –* 45-year-old unaffected female, *BRCA2* PV/LPV carrier.

## Discussion

This interview study aimed to explore the post-result informational needs of *BRCA1/2* PV/LPV carriers and how best to fulfill them. This study identified unmet informational gaps which led to challenges in GT result disclosure and risk management decision-making. It has resulted in proactive online information-seeking behaviors. There is a desire for more reliable sources of online information with personalized, local content. All participants perceive the general lack of genetic awareness as a core shortcoming of the system, and if alleviated, would be able to ease the multi-faceted challenges they face as carrier.

Our findings preliminarily demonstrate that the probands’ lack of personal understanding and informational support leads to challenges in disclosing their GT result to at-risk family members. Similarly, Montgomery et al. [29] and Vavolizza et al. [30] found that probands cite a poor understanding and an inability to explain the implications of their result as reasons for the reluctance to disclose. Our participants further perceive that the challenges they face with GT result disclosure could be alleviated with additional online sources of information that can be easily shared with family members to support result disclosure discussions.

Notwithstanding, we observed a cohesive underlying desire for online informational support from participants, to support personal understanding, result communication, and risk management decisions. Their desire for information has perpetuated into proactive online information-seeking behaviors demonstrated by carriers of all ages. This is in contrast to the findings of previous studies in USA and Australia [31, 32], where carriers were less proactive, and more inclined to only receive information in-person from clinicians. Our findings seem to suggest that *BRCA1/2* PV/LPV carriers in Asia tend to be information-seeking, internet-savvy, preferring information in the form that they can access or share. It further highlights the importance of supporting these online information-seeking behaviors, where the provision of video- and/or online-based information could be well-received by *BRCA1/2* PV/LPV carriers in Asia.

Aligned with our participants preferences for convenient, accessible and online sources of information – intrafamilial conferences facilitated through telehealth services could be a feasible model to foster better disclosure experiences, where discussions could focus on the actionable implications of the GT result for at-risk relatives. In the long run, this could mitigate the low rates of disclosure that underlie poor uptake of predictive testing [33, 34], which has been identified as an obstacle preventing the current healthcare system from reaping the cost-efficiencies of genetic testing [23].

Participants also revealed strong preferences for content to be provided in a personalized manner, particularly based on age. Our findings add to existing literature that young female *BRCA1/2* PV/LPV carriers face a unique set of challenges as compared to post-menopausal women: reduced sexuality and libido [35], a compressed family cycle [36] and more quality of life changes post-RRBSO [37]. In our study, these concerns have been highlighted as difficult, verging on embarrassing, to raise with managing clinicians – and hence have largely gone unanswered, suggesting the utility of decisions aids [38, 39] in addressing these hard-to-raise informational gaps.

Similarly, young participants have voiced a need for more age-appropriate materials to support the understanding of their pre-adult children. These views are consistent with other studies [40], where sharing and non-sharing mothers expressed that their greatest need in relation to GT result disclosure was child-specific, age-appropriate genetic information, which emphasizes the positive, preventive utility of genetic information to their minor children. The desire for a formal informational and psychosocial support system also stemmed from younger participants; who saw value in having a designated companion to discuss the challenges they face as *BRCA1/2* PV/LPV carriers. A similar model, of telephone-based peer support between pairs of *BRCA1/2* PV/LPV carriers [41], observed that while satisfaction with peer-provided support was high, 80% of peer support-pairs ended contact amicably, largely due to age- and surgery experience-incompatibility between pairs. These findings are consistent with participant feedback that such formal support systems could be optimized if patient companion pairs are matched by age and/or affected status.

Participants hoped for the provision of content to personalized and culturally-relevant, especially in the form of patient stories. In keeping with our participants’ desire for online information, they similarly hope to see the utilization of online social media platforms [42], to facilitate the sharing of local patients’ stories for greater publicity – their suggestions are perhaps an intention to trigger a wave of the Angelina effect [43] locally, de-identifying the *BRCA1/2* PV/LPV status as a Hollywood or *“Caucasian*” disease. Furthermore, digital storytelling is a proven tool for health promotion and cancer awareness [44], and when incorporated with social media, could produce desirable effects on community-wide genetics education [45, 46].

Interestingly, there was a united appeal for greater genetic awareness as a solution to mediate several challenges they face as a carrier, which include poor familial understanding of their GT result [29]; insurance discrimination [47]; lack of genetic testing cost subsidies [23]; stigmatization they face as *BRCA1/2* PV/LPV carrier [48]. Cost has long been a barrier to genetic testing uptake in Asia [49], often with significant out-of-pocket costs borne by patients – a result of the absence of genetic testing adoption into the public healthcare system [50]. In an effort to reduce insurance discrimination, there have been genetic non-discrimination laws enacted to protect carriers in Europe, America, and Australia, whilst most Asian countries have yet to follow suit [51]. While existing models generally suffer inefficiencies due to limited public visibility and poor genetic literacy, legislation is still necessary to target genetic discrimination. The lack of genetic awareness and poor genetic literacy is cited as a problem even amongst the educated [52], and likely arises from the lack of readily available resources providing clear and accurate genetic information to the public. It illustrates the need for an educational genetics platform, catered not only to carriers, but actively engaging the general public, integrating genetics into everyday life – to alleviate the struggles that carriers face.

### Study limitations

This study should be considered in light of certain limitations inherent to qualitative research. We acknowledge that our study may lack representation of non-English speaking minority groups, as our cohort was limited to English-speaking participants. It was designed in this manner as the majority (79%) of the population speak English [41]. Additionally, we were unable to conduct non-English interviews due to the inability of the interviewer to speak other languages, and the lack of resources to translate non-English transcripts into English. Such a form of ascertainment bias may have inadvertently overestimated the degree of information-seeking behaviors within our population; however, this is predicted to be minimal as our selected participant group is largely reflective of the entire population. Considering the high rates of digital literacy in Singapore, our findings may not be reflective of *BRCA1/2* PV/LPV carriers of other populations who may not share the same levels of digital literacy.. Our participant cohort was largely female, indicating a further need to understand if the informational needs of male carriers coincide with female carriers. Additionally, it would be meaningful to study if provision of information should be personalized based on other demographics such as the family history of cancer, ethnicity and literacy level.

Nonetheless, our findings guide researchers to focus on issues raised by *BRCA1/2* PV/LPV carriers that may pose as barriers in maximizing their cancer risk management options that arise with the knowledge of their *BRCA1/2* status. Future studies could explore the use of different models and/or platforms (e.g. patient stories, telehealth, social media) to support the informational needs and online-seeking behaviors of *BRCA1/2* PV/LPV carriers.

## Conclusions

Participants highlight challenges with sharing their GT result arising from significant post-result informational needs, which have manifested into proactive online information-seeking behaviors. They demonstrated a desire for an online source of information provision, where content is personalized, reliable and local, preferably supported with patient stories. They hope that the availability of such information resources can raise genetic awareness among broader society. This suggests the use of a culturally tailored online-based resource, to address knowledge gaps and raise genetic literacy rates, may be successful for addressing these unmet needs.

## Data Availability

The datasets used and/or analyzed during the current study are available from the corresponding author on reasonable request.

## Addditional file

**Additional file 1.**

## Abbreviations

GT: Genetic test
PV/LPV: Pathogenic variant/ likely pathogenic variant
RR: Risk-reducing
RRBSO: Risk-reducing bilateral salpingo-oophorectomy
CGS: Cancer genetics service
NCCS: National Cancer Centre Singapore
