# Supplementary material for: An in-depth exploration of the post-test informational needs of BRCA1 and BRCA2 pathogenic variant carriers in Asia
Source: Hered Cancer Clin Pract. 2020 Oct 23;18:22. doi: 10.1186/s13053-020-00154-x (PMC7585213; doi:10.1186/s13053-020-00154-x)
Supplement: Supplementary file 1 — Additional file 1. [file 13053_2020_154_MOESM1_ESM.pdf]

<u>Supplementary Materials 1: Interview Guide</u>

Research Question: What are the informational needs of patients who carry a *BRCA1/2* pathogenic variant?

Thank you for participating in this interview. The goal of this research study is to understand what information would be helpful to people like yourself, who have been found to have a faulty BRCA1 or BRCA2 gene (associated with hereditary breast and ovarian cancer syndrome). Your responses today will help guide the content of a video we are creating that is meant to support people who have received a genetic result similar to yours, and may require more support or information to make medical decisions.

I would like to let you know that the interview will be audio-recorded in order for us to not miss any valuable information discussed. Just to assure you, everything you say during this interview would be kept confidential and your responses will be de-identified and kept anonymous.
Before we begin, I would like to ask you to your cell phone to silent or vibrate.

<u>Segment 1: Exploring patients' experiences when receiving their results from a genetic counsellor</u>

We would like to start out by asking you to think back to the time you received your genetic result from your genetic counsellor.

1) What do you remember from your result session with your genetic counsellor?

Probes:

- What do you understand about your genetic result?
- What can you tell us about what your genetic counsellor told you?
- What was the most helpful piece of information you received from that session?
- What do you think it means to have a faulty *BRCA1/2* gene?
- How did you feel when you received your genetic result?

2) What does your family understand about the genetic result you received?

Probes:

- How did you share your result with your family?
- What information did you share with your family, if any?
- What (information) influenced your decision to share your result with your family?
- What information was helpful in sharing the result with your family?
- What were some of their thoughts after hearing your result?
- How did your family react after hearing about your result?

3) What other information would you have liked to receive from your genetic counsellor?

Probes:

- What information did you not receive that could be helpful for other people in a similar situation?
- What other information would you or your family members' find helpful to receive?
- What questions did you have after your result session with your genetic counsellor?
- What questions did your family members have after you shared your result with them?
- What other information could be helpful to you in explaining your result to your family?

Segment 2: Exploring patients' experiences with specialists regarding medical decisions

You may have been referred to specialised doctors to discuss different ways you can reduce your risk of cancer. You may have decided on a way you would like to manage your risk of cancer, be it through screening or surgery in certain cases. We would like to know what information played a role in helping you make this decision, or may assist with future decisions you may need to make.

4) What doctors were you referred to following your result?

- Which doctors did you discuss your result with?

5) What do you remember from your session(s) with the ____ doctor(s) whom you were referred to?

Probes:

- What information did you receive?
- What did the doctor explain to you?

- What was discussed during that consultation?
- What did you understand from your session with the doctor?
- How did you feel about the information you received from your doctor?

6) You may have been faced with the decision to undergo screening or surgery. What information would you find useful in helping others (like yourself) make such decisions?

Probes:

- What information do you think plays a role in deciding between screening or surgery?
- What discussions with your doctor were helpful in deciding between screening or surgery?
- What do you think people would consider when making decisions like this?
- What questions did you/do you have regarding making this decision?

7) Thinking back to your discussions with your _____ doctor, what other information would you have liked to receive?

Probes:

- What information did you not receive that could be helpful for other people in a similar situation?
- What questions did you have after your session with your doctor?

Segment 3: Exploring patients' needs for informational support and resources

Following receiving your genetic result, we would like to be able to support you and your family members through the process of understanding what the genetic result means for you and your family.

7) In what ways would we (as a service) be able to better support you?

Probes:

- What information would you like to receive about other support resources we can provide?
- In what other ways would you like to receive support from us?
- What other information could be useful in supporting other people's knowledge about their results?

8) From your experience, what tips and insights would you like to share with other people who are going through a similar experience?

We have come to the end of all the questions. Thank you for sharing your insights with us. Your responses are very valuable and will help us provide the best care possible to you and other people in similar situations as you.

Before we end, does anyone else having anything further they would like to share with us which we have not covered?

Feedback Segment (only for pilot interviews)

- Do you have any comments/feedback that you would like to give regarding this interview?
- Was there any question that was confusing/difficult to understand?
- Did you find any question offensive?
- Do you have any feedback regarding the moderator?
- Can you think of any questions which we did not ask but that you think would be important?
